# Assessing the risk of human trichinellosis from pigs kept under controlled and non-controlled housing in Europe

**DOI:** 10.1016/j.fawpar.2018.04.003

**Published:** 2018-04-19

**Authors:** Frits Franssen, Katsuhisa Takumi, Joke van der Giessen, Arno Swart

**Affiliations:** National Institute for Public Health and the Environment (RIVM), Bilthoven, The Netherlands

**Keywords:** Trichinellosis, QMRA, Controlled housing, Non-controlled housing, Meat inspection

## Abstract

To support risk-based approach to prevent human trichinellosis, we estimated the human incidence for pigs originating from controlled and non-controlled housing, using a quantitative microbial risk assessment model for *Trichinella* (QMRA-T). Moreover, the effect of test sensitivity on human trichinellosis incidence from pigs from non-controlled housing was quantified. The estimated annual risk from pigs from non-controlled housing was 59,443 human trichinellosis cases without testing at meat inspection and 832 (95%CI 346–1410) cases with *Trichinella* testing, thus preventing 98.6% of trichinellosis cases per year by testing at meat inspection. Using the QMRA-T, a slight decrease in test sensitivity had a significant effect on the number of human trichinellosis cases from this housing type. The estimated annual risk for pigs from controlled housing was <0.002 (range 0.000–0.007) human cases with- and <0.010 (0.001–0.023) cases without *Trichinella* testing at meat inspection, which does not differ significantly (*p* = 0.2075). In practice, this means no cases per year irrespective of *Trichinella* testing. Thus controlled housing effectively prevents infection and *Trichinella* testing does not contribute to food safety for this housing type. Not testing for *Trichinella* requires evidence based full compliance with regulations for controlled housing.

## Highlights

• Controlled housing effectively prevents *Trichinella* infection risk for humans.
• Testing pigs from controlled housing at slaughter does not add health benefits.
• Not testing requires evidence based compliance with controlled housing regulations.
• Testing pigs from non-controlled housing prevents 98.6% of human trichinellosis cases.
• The residual risk of human trichinellosis is 832 cases per year for the EU.

## 1 Introduction

Human trichinellosis is caused by ingestion of *Trichinella* muscle larvae in raw or improperly cooked meat or meat products. Within the nematode genus *Trichinella*, twelve taxa are recognized, which can infect a wide range of carnivores and omnivores, including humans and pigs (Pozio et al., 2009; Pozio and Murrell, 2006; Pozio and Zarlenga, 2013).

In Europe, the control of *Trichinella* is laid down in EU regulation 2015/1375 (European-Commission, 2015) for pigs, horses and other *Trichinella* susceptible animals used for human consumption. Biosecure or controlled housing conditions are directed at prevention of exposure to *Trichinella* and have to comply with regulations addressing feed safety, rodent control, building requirements, recorded management practices and on site audit programs (European-Commission, 2015; FAO-WHO, 2014a). Private standards such as the Danish Product standard in Denmark, QS Qualität und Sicherheit GmBh in Germany and Integrale Ketenbeheersing (IKB) in the Netherlands have been put in place to perform regular on-site audits and to document compliance with national law, and customer- and trade partner requirements (Alban and Petersen, 2016).

Worldwide, >50% of slaughter pigs are produced under bio-secure (controlled) housing conditions (Pozio, 2014). Approximately 120 million (75%) of fattening pigs in Europe are kept under controlled housing conditions (Pozio, 2014) and none of these animals tested *Trichinella*-positive during the past two decades (Alban et al., 2008; Alban et al., 2011; Pozio, 2014). Consequently, the benefits of the *Trichinella* control program by testing pig carcasses at slaughter do not support the costs for these pigs. Therefore, *Trichinella* control moved to a risk-based approach and an exemption is made in EU legislation for pigs that are kept under controlled housing conditions, which means that these pigs no longer need to undergo individual carcass control (European-Commission, 2015).

To support this risk-based approach, we developed a quantitative microbial risk assessment model for *Trichinella* (QMRA-T) that includes the whole chain of events from primary production up to human trichinellosis incidence, based on prevalence data of pigs from non-controlled housing and wild boars, in combination with incidence data for human trichinellosis (Franssen et al., 2017). Defining parameters to estimate residual *Trichinella* infection risk for pigs from controlled housing in the QMRA-T is not straightforward, since no *Trichinella* positive animals have been demonstrated at meat inspection.

*Trichinella* testing at meat inspection is performed using a standardized reference method (European-Commission, 2015; ISO, 2015; Rossi and Pozio, 2008), for which test characteristics have been evaluated extensively (Riehn et al., 2013; Vallée et al., 2007). Recently, alternative tests have been introduced onto the market that may not have the same sensitivity as the reference method. So far, the effect of test sensitivity on the resulting number of human trichinellosis cases has not been quantified.

In the present study, we quantitatively evaluated the infection risk from pigs reared under controlled and non-controlled housing conditions with- and without *Trichinella* testing at meat inspection, using the QMRA-T model. Moreover, the effect of test sensitivity on expected human trichinellosis incidence from pigs from non-controlled housing was assessed.

## 2 Materials and methods

We used the QMRA-T model parametrized with data for pigs from non-controlled housing and wild boar from Poland, to model risk expressed as expected number of human trichinellosis cases per year (which may be a fractional number) (Franssen et al., 2017). Furthermore, the model performance was favorably assessed by comparing with epidemiological estimates of human cases. Briefly, the model used parameters to determine larval distribution between and within animals from a negative binomial distribution, followed by calculation of the number of *Trichinella* larvae per 100 g of diaphragm and probability of sampling larvae with 1 g (or 5 g for wild boar) diaphragm sample size from a binomial distribution at meat inspection. Next, the probability of recovering larvae from a pool of 100 g diaphragm samples following a beta-binomial distribution was determined. Missed positive carcasses at meat inspection and the number of *Trichinella* larvae in such positive carcasses are registered in the model. Distribution of *Trichinella* larvae over portions of pork was determined using a multinomial distribution. Finally, *Trichinella* inactivation after cooking was modelled based on consumer preference literature data, which were compared to and combined with temperature inactivation data from the literature. A previously described *Trichinella* dose-response for human infection (Teunis et al., 2012) was included in the QMRA-T, resulting in the number of human trichinellosis cases. One model run performed 1000 simulations with each simulation representing 1 year, to obtain robust estimates. Hence, model results are presented as mean with 95% confidence intervals (CI) and variability over model runs represents variability between years.

### 2.1 Model parameters

Table 1 shows all parameters used for the present QMRA. Moreover, an extra module was added to evaluate *Trichinella* test sensitivity at meat inspection, introducing an additional parameter named “sens”. Finally, a variable “max larvae” was introduced, which limited the maximum number of larvae per model run, to explore the number of larvae per 100 g (LP100G) in the diaphragm of pigs from controlled housing.

**Table 1:** Test parameters for QMRA modelling of human trichinellosis cases for non-controlled and controlled housing with- and without *Trichinella* testing at meat inspection.

Variable parameter	Pig, non-controlled	Pig, controlled	Unit
Observed prevalence	5.26 × 10^−6^	<4.17 × 10^−10^	–
*m*	6.87 × 10^−3^	5.30 × 10^−7^	–
*k*	5.83 × 10^−7^	4.51 × 10^−11^	–
Abundance	0.3–211	0.1–0.8	LPG
*Trichinella* relative test sensitivity^a^	0.6–1 and 0	1 and 0	–
Number of swine	80	120	Million/year
Swine/pool	100		Pigs
Diaphragm weight tested	1		Gram
Iterations # escaped swine	1000		
Iterations # larvae in diaphragm	1000		–
Portions/person^b^	94	147	100 g
Population EU^c^	504		Million
Number of loops per model run	1000		–

*m*: mean number of *Trichinella* muscle larvae (ML) in 50 g of diaphragm.

*k*: the clustering of *Trichinella* ML among individual swine.

a Test sensitivity relative to the sensitivity of *Trichinella* testing using the artificial digestion test. A relative test sensitivity of 1 means testing according to the EU Reference method, 0 means no testing at all.

b Average consumption of portions of shoulder, loin and belly per person per year calculated for EU, proportional to housing condition of origin.

c Population size EU 2015; average EU population 2007–2016: 503.5 ± 3.2 M (Table 4).

### 2.2 Pigs from controlled housing

EU member states are obliged to report *Trichinella* test results at meat inspection to the European Food Safety Authority (EFSA) annually, including data on pig type (breeding or fattening), housing conditions and the number of pigs that tested positive for *Trichinella.* Before 2012, housing conditions were not reported to EFSA; human trichinellosis cases are reported to the European Centre for Disease Control (ECDC). Data from both humans and animals are combined in annual EFSA-ECDC reports on zoonoses, zoonotic diseases and food-borne outbreaks. Data from these reports over the years 2012–2015 were compiled to determine numbers of pigs from controlled housing that are slaughtered annually in the EU (EFSA-ECDC, 2010a, EFSA-ECDC, 2013, EFSA-ECDC, 2014, EFSA-ECDC, 2015, EFSA-ECDC, 2016). The annually reported data of pigs were corrected for countries that keep (virtually) all pigs under controlled housing but did not report this explicitly (NL, DK).

During the period 2012–2015, 61% of slaughtered pigs (442,290,621 out of 725,672,360 in total, both breeding and fattening pigs) had been kept under controlled housing and this proportion was extrapolated to the period 2007–2011 to estimate the number of pigs from controlled housing during the latter period. Over the years 2007–2015, on average 195 ± 22.6 million pigs have been slaughtered annually in the EU, of which on average 119 ± 14.8 million pigs (61%) originated from controlled housing (Table 2). This estimate was used to calculate an upper prevalence limit for *Trichinella* in those pigs. On average 78.9 ± 12.5 million pigs that are slaughtered annually originate from non-controlled housing and unspecified housing combined (Table 2).

**Table 2:** Overview of number of *Trichinella* tested and positive pigs per housing type over the years 2007–2015 in the EU. Data compiled from EFSA-ECDC reports 2008–2016 (EFSA-ECDC, 2009, EFSA-ECDC, 2010a, EFSA-ECDC, 2010b, EFSA-ECDC, 2013, EFSA-ECDC, 2014, EFSA-ECDC, 2015, EFSA-ECDC, 2016). Controlled: number of slaughtered pigs from controlled housing. Non-controlled: number of slaughtered pigs from non-controlled housing and non-specified housing combined.

Year	Controlled	Trich-pos	Non-controlled	Trich-pos^a^	Prevalence	Totals
2007	134,615,018	0	86,065,340	728	8.46 × 10^−6^	220,680,358
2008	132,714,386	0	84,850,182	1179	1.39 × 10^−5^	217,564,568
2009	123,158,444	0	78,740,645	430	5.46 × 10^−6^	201,899,089
2010	128,940,766	0	82,437,539	199	2.41 × 10^−6^	211,378,305
2011	109,300,558	0	69,880,685	304	4.35 × 10^−6^	179,181,243
2012	127,800,046	0	80,344,483	331	4.12 × 10^−6^	208,144,529
2013	94,182,495	0	60,215,037	363	4.26 × 10^−6^	154,397,532
2014	98,562,603	0	92,781,250	208	2.24 × 10^−6^	191,343,853
2015	121,745,478	0	50,040,968	106	2.12 × 10^−6^	171,786,446
Totals	1,071,019,795		710,423,804	3848	–	1,756,375,923
Average	119,002,199	0	78,935,978	428	5.26 × 10^−6^	195,152,880
SD	14,835,254	0	12,494,905	333	3.79 × 10^−6^	22,660,773
Median	123,158,444	–	82,437,539	331	4.26 × 10^−6^	201,899,089

a Number of *Trichinella* positive pigs from non-controlled and non-specified housing combined. Prevalence: *Trichinella* prevalence per year. Totals: total number of pigs slaughtered and tested for *Trichinella* in a given year.

### 2.3 *Trichinella* upper prevalence limit in pigs from controlled housing

The annual number of 120 million pigs from controlled housing was used to calculate an upper prevalence limit for *Trichinella* in those pigs, to allow computations for this housing type. During the last two decades, none of the pigs from controlled housing in the EU tested positive for *Trichinella* (Pozio, 2014). Consequently, this resulted in 0 out of 2.4 billion (20 × 120 million) pigs from controlled housing being positive for *Trichinella*, yielding an upper prevalence estimate $U<4.17\times 10^{-10}\left(=\frac{1}{2.4\times 10^{9}}\right)$.

### 2.4 *Trichinella* larvae distribution and abundance in pigs from controlled housing

In the QMRA-T, prevalence is defined by the parameters of a negative binomial distribution: *m* (mean number of *Trichinella* muscle larvae in 50 g of diaphragm) and *k* (the clustering of *Trichinella* muscle larvae among individual swine). Two 50 g diaphragm portions make up one 100 g portion, which is the sum of two negative binomial variables, which again is a negative binomial distribution with parameters 2 *m* and 2 *k*. Throughout the model 100 g consumer portions were calculated using these parameters (Franssen et al., 2017). Lacking data from *Trichinella* positive pigs from controlled housing, we empirically determined pairs of values for *m* and *k* by random sampling. Several combinations of logical ranges for *m* and *k* were tested, and the combination *m* = 5.3 × 10^−7^ and *k* = 4.5 × 10^−11^ yielded a prevalence (4.2 × 10^−10^) that matched the upper prevalence limit of 4.17 × 10^−10^ and therefore these parameters were used in the QMRA to model trichinellosis risk from pigs housed under controlled conditions (Table 1).

**Fig. 1:**
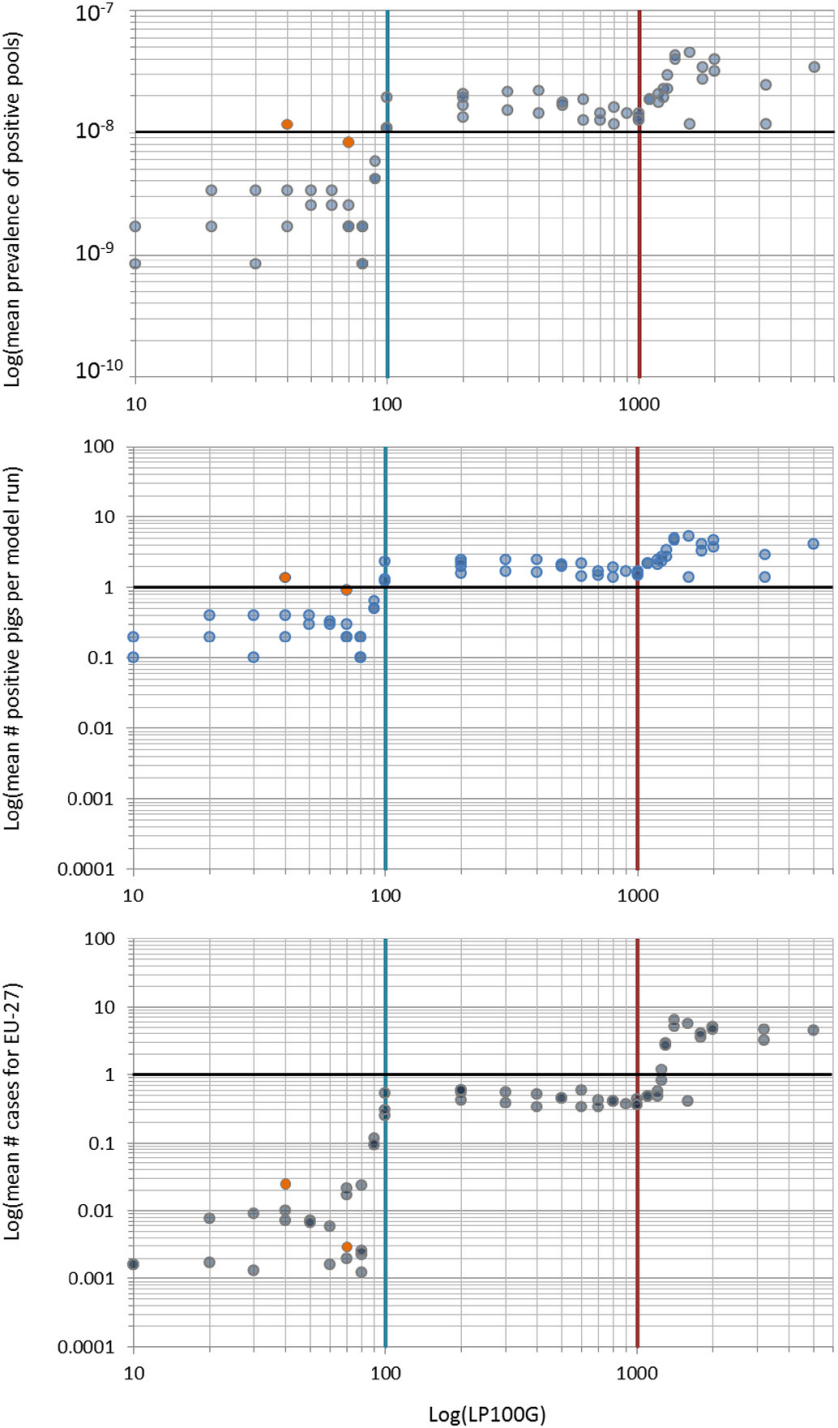
Hypothetical *Trichinella* abundance in pigs from controlled housing based on scenario analysis. To find a level of *Trichinella* larval abundance that matched the upper prevalence limit of 4.17 × 10^−10^ for pigs from controlled housing, QMRA-T model runs were performed with increasing numbers of larvae per model run, ranging 10–5000 *Trichinella* larvae per 100 g of diaphragm. Each dot represents one model run. Orange dots represent model runs with increased sample size (5 g instead of 1 g). 1A. Prevalence of positive pools per model run at increasing numbers of *Trichinella* larvae per 100 g diaphragm (LP100G) on a logarithmic scale are shown. In the range 10–80 LP100G, the average number of positive pools ranged 0.9 × 10^−9^–3 × 10^−9^. Black horizontal line represents 1 positive pool. 1B. Corresponding numbers of positive pigs ranged 0.1–0.3 per positive pool, resulting in an overall prevalence ranging 0.9 × 10^−10^–9 × 10^−10^ (prevalence of positive pools × prevalence of positive pigs in those pools), which fitted a hypothetical upper prevalence limit of 4.2 × 10^−10^ for pigs from controlled housing. All other combinations resulted in prevalence values above this upper prevalence limit. Black horizontal line represents 1 positive pig in a positive pool. 1C. Mean number of human trichinellosis cases for the EU corresponding with positive pools and positive pigs in those pools. Black horizontal line represents 1 human trichinellosis case.

Subsequently, scenario analyses were performed using the empirically determined parameters *m* and *k*, and increasing maximum numbers of larvae per 100 g (LP100G), to determine the most plausible corresponding number of larvae that fitted the upper prevalence limit. At least two model runs were performed per LP100G level, resulting in 70 model runs with “max larvae” values ranging from 10 to 5000 on a Log scale (on average 0.1–50 LPG). Running the QMRA-T, only a range of 10–80 *Trichinella* larvae per 100 g (0.1–0.8 LPG) resulted in a prevalence range (0.9 × 10^−10^–9 × 10^−10^) that matched the upper prevalence limit mentioned above (Fig. 1). Therefore, the model parameters were set at “max larvae” = 80, *m* = 5.3 × 10^−7^ and *k* = 4.5 × 10^−11^ to model pigs from controlled housing.

### 2.5 *Trichinella* larvae distribution in pigs from non-controlled housing

Over the years 2007–2015, in total 3848 pigs out of 710,423,804 from non-controlled housing tested positive for *Trichinella* in the EU (EFSA-ECDC, 2009, EFSA-ECDC, 2010a, EFSA-ECDC, 2010b, EFSA-ECDC, 2013, EFSA-ECDC, 2014, EFSA-ECDC, 2015, EFSA-ECDC, 2016) (Table 2). Using these data and observed *Trichinella* larval abundance (0.3–211 LPG) described in a preceding publication of the QMRA-T (Franssen et al., 2017), *m* and *k* were estimated for this population at 6.872 × 10^−3^ and 5.828 × 10^−7^ respectively.

### 2.6 Per capita pork consumption for Europe

The per capita pork consumption data used in this study are based on the Balance of Pork Production, Import and Export, in relation to population size for the EU over the years 2008–2015 (AHDB, 2016). The number of portions of shoulder, loin, and belly (the parts that consumers buy raw and cook at home) has been estimated previously at 59% of the total number of portions per carcass (Franssen et al., 2017). The overall annual consumption of pork of twelve selected EU member states was on average 40.8 ± 0.7 kg (range 24–56 Kg) or on average 408 portions of 100 g pork per person (AHDB, 2016). Applying the proportion of shoulder, loin and belly per carcass resulted in on average 241 ± 4 portions of these parts combined, per EU citizen per year (Table 1). Overall in the EU, 147 (61%) of all yearly consumed portions would originate from pigs that had been kept under controlled housing and 94 portions from pigs that had not been kept under controlled housing. We assumed in these calculations a full mixing of portions in the market.

### 2.7 Relative test sensitivity at meat inspection

Both controlled and non-controlled housing types were modelled with- (“sens” = 1) and without *Trichinella* testing (“sens” = 0) at meat inspection. Earlier, the test sensitivity (the probability to find at least one *Trichinella* muscle larva in a sample, given the number of larvae present) had been determined at 0.843–0.991 for one up to ten *Trichinella* larvae present in a 100 g diaphragm sample (Franssen et al., 2017), using the EU Reference method (European-Commission, 2015), which is regarded the gold standard. To evaluate the influence of test sensitivity at meat inspection on the number of human trichinellosis cases, we monitored the number of missed *Trichinella* positive carcasses from non-controlled housing at relative test sensitivities of 85–100% compared to the gold standard. Three QMRA-T model runs were performed for each relative test sensitivity evaluated. The resulting number of human trichinellosis cases at decreased test sensitivity was recorded. A trend line was fitted through the data points using Microsoft Excel to characterize the relative risk as *RR* = *a* × *%Sensitivity reduction* + *Base line sensitivity*.

### 2.8 Statistical analysis of model outcomes

For controlled housing, we estimated human trichinellosis cases with- and without testing from five model runs each and 1000 loops per model run, significance of outcome difference was tested using a paired *t*-Test in Microsoft Excel.

## 3 Results

### 3.1 Human trichinellosis risk from pigs from non-controlled housing

Using reported consumption data and assuming proportional consumption of pork from non-controlled housing (94 out of 241 annually consumed portions/person), the estimated risk of human trichinellosis from pigs from non-controlled housing in the EU was estimated at 832 (95%CI 346–1410) cases per year or 1.65 (95%CI 0.68–2.79) cases per million per year after testing for *Trichinella* (Table 3). This result is close to on average 515 annually reported trichinellosis cases, of which 409 have been confirmed with laboratory tests (EFSA-ECDC, 2009, EFSA-ECDC, 2010a, EFSA-ECDC, 2010b, EFSA-ECDC, 2013, EFSA-ECDC, 2014, EFSA-ECDC, 2015, EFSA-ECDC, 2016) (Table 4). Without *Trichinella* testing at meat inspection, the number of human trichinellosis cases was estimated at 59,443 (95%CI 52,837–66,360) per year. From these results, the benefits of *Trichinella* testing at meat inspection for non-controlled housing were estimated at 98.6% reduction of trichinellosis cases per year.

**Table 3:** Effects of housing condition and *Trichinella* testing on the number of annual human trichinellosis cases. The QMRA-T was used to calculate the annual number of human trichinellosis cases from pigs form non-controlled and controlled housing with- and without *Trichinella* testing at meat inspection.

	Non-controlled, tested		Non-controlled, not tested		Controlled, tested	Controlled, not tested
	Mean	95%CI	Mean	95%CI	Mean (range)^1^	Mean (range)^a^
A. False negative pools	2.61 × 10^−5^	1.52 × 10^−5^–3.74 × 10^−5^	5.58 × 10–4	5.08 × 10^−4^–6.07 × 10^−4^	8.40 × 10^−10^ (0.00–2.50 × 10^−9^)	1.34 × 10^−9^ (8.40 × 10^−10^–1.68 × 10^−9^)
B. Positive carcasses in those pools	1.00	1.00–1.00	1.00	1.00–1.00	0.00–0.01	0.01 (0.01–0.01)
C. Illness per million portions	1.76 × 10^−2^	7.30 × 10^−3^–2.97 × 10^−2^	1.26	1.26–1.40	2.65 × 10^−8^ (0.00–9.76 × 10^−8^)	1.41 × 10^−7^ (1.68 × 10^−8^–3.18 × 10^−7^)
D. Consumptions of 100 g per year	94		94		147	147
E. Average EU population size 2007–2016	5.04 × 10^8^		5.04 × 10^8^		5.04 × 10^8^	5.04 × 10^8^
F. Total consumed portions	4.73 × 10^10^		4.73 × 10^10^		7.40 × 10^10^	7.40 × 10^10^
G. Total predicted human cases per year	832 (346–1410)		59,443 (52,837–66,360)		<0.002 (0.000–0.007)	<0.01 (0.001–0.023)
H. Predicted human cases/million/year	1.65 (0.68–2.79)		118 (105–132)		<3.90 × 10^−6^ (0.00–1.44 × 10^−5^)	<2.07 × 10^−5^ (2.47 × 10^−6^–6.67 × 10^−5^)

A: average number of pools that escaped *Trichinella* detection at meat inspection containing 1 g diaphragm samples each of 100 animals. B: the average number of positive pigs in those pools of 100 animals. C: average number of *Trichinella* infected portions per million portions of shoulder, loin and belly combined. D: average annual pork consumption per person in the EU, proportional to housing type. A full mixing of portions from both housing types was assumed in the market.

a Average value of five separate model runs repeated 1000 times each.

**Table 4:** Reported cases of human trichinellosis in the EU over the years 2007–2015. Data compiled from Eurostat and EFSA-ECDC reports 2008–2016 (EFSA-ECDC, 2009, EFSA-ECDC, 2010a, EFSA-ECDC, 2010b, EFSA-ECDC, 2013, EFSA-ECDC, 2014, EFSA-ECDC, 2015, EFSA-ECDC, 2016).

Year	Population (M)	Cases (tot)	Incidence/M	Cases (conf)	Incidence/M
2007	498.3	867	1.740	780	1.565
2008	500.3	680	1.359	670	1.339
2009	502.1	1073	2.137	748	1.490
2010	503.2	394	0.783	223	0.443
2011	503.0	363	0.722	268	0.533
2012	504.1	378	0.750	301	0.597
2013	505.2	256	0.507	217	0.430
2014	507.0	383	0.755	319	0.629
2015	508.5	243	0.478	156	0.307
Average	503.5	515	1.026	409	0.815
SD (range)	3.2	(243–1073)	0.584	(156–780)	0.500

Population (M): population size in millions for a given year. Cases (tot): total number of reported trichinellosis cases based on clinical signs. Cases (conf): number of human trichinellosis cases confirmed by laboratory analysis. Incidence/M: incidence per million EU inhabitants.

### 3.2 Human trichinellosis risk from pigs from controlled housing

Using reported EU consumption data, assuming proportional consumption of pork from controlled housing (147 out of 241 portions), with *Trichinella* testing at meat inspection, the estimated annual number of human trichinellosis cases was on average < 0.002 (range 0.000–0.007) for the whole EU (Table 3). Without testing, the estimated annual number of trichinellosis cases was <0.010 (range 0.001–0.023) per year (Table 3), which did not differ significantly from the outcome with *Trichinella* testing (*p* = 0.2075, paired *t*-Test). In practice, this means no cases per year both with- and without *Trichinella* testing.

### 3.3 Relative test sensitivity at meat inspection

**Fig. 2:**
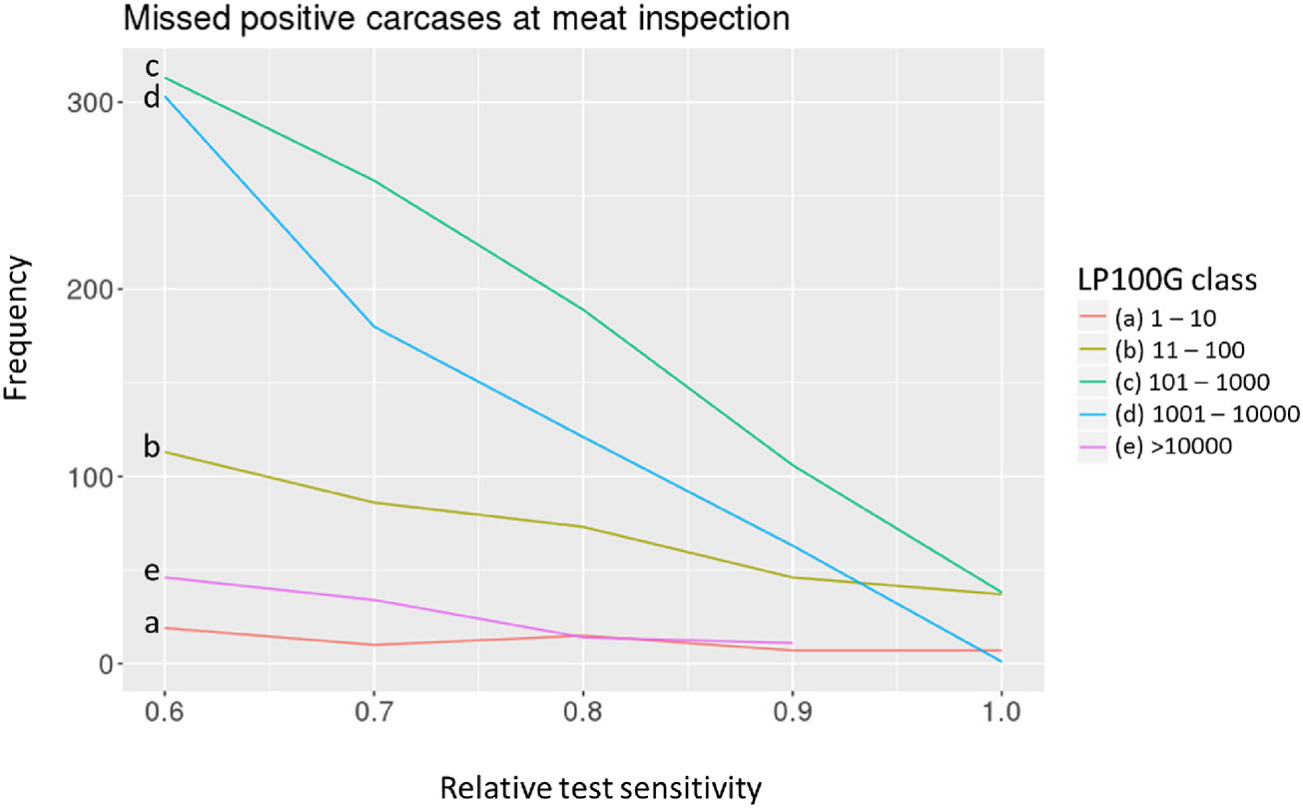
Frequency of missed positive carcasses at meat inspection at varying sensitivity using the artificial digestion test. At larval abundance above 1000 *Trichinella* muscle larvae per 100 g of diaphragm (LP100G) (d, e), the frequency of missed positive carcasses decreased to zero at maximum relative test sensitivity (1.0). At lower infection levels per 100 g diaphragm (a, b, c), the frequency of missed positive carcasses decreased with increasing test sensitivity. However, due to a test sensitivity limit of 1 LPG (100 LP100G) of the artificial digestion test at meat inspection, not all positive carcasses will be detected.

At larval abundance above 1000 *Trichinella* muscle larvae/100 g (on average 10 LPG, Fig. 2d and e) in the diaphragm of pigs kept under non-controlled housing, the frequency of missed positive carcasses decreased to zero at relative test sensitivity equal to the EU reference method. At lower abundance levels (Fig. 2a, b, c), positive carcasses will be missed at meat inspection, even at 100% relative test sensitivity. At lower relative test sensitivity, the frequency of missed positive carcasses was considerably higher, even at high larval abundance (Fig. 1c, d). Extremely low or high larval abundance occurred at such low frequency that relative test sensitivity had little influence on the frequency of missed positive carcasses (Fig. 2a and e).

**Fig. 3:**
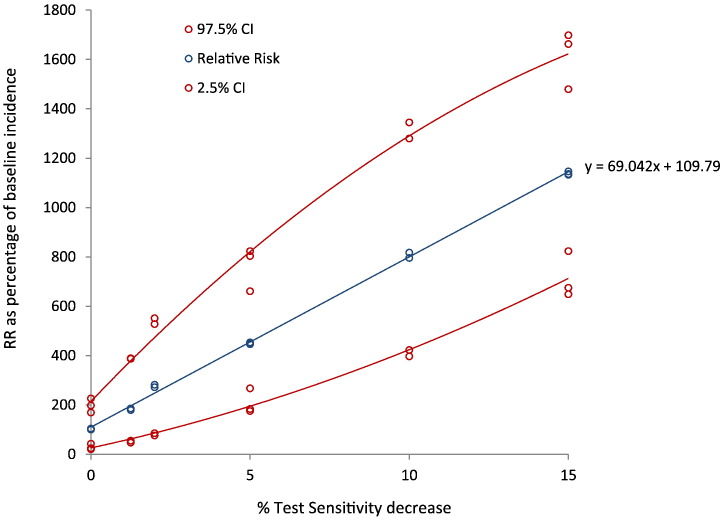
Number of annual trichinellosis cases in relation to varying relative test sensitivity using the EU reference method. The actual risk of human trichinellosis was modelled using the QMRA-T for pigs from non-controlled housing, for test sensitivity ranging 85–100% of gold standard at meat inspection. The blue line shows the regression line through three replicate model runs at each relative sensitivity value tested. The red lines show the upper (97.5%) and lower (2.5%) confidence limit for each model run. RR: relative risk. Baseline incidence is 100%.

A slight decrease in test sensitivity at meat inspection will have a significant effect on the increase of estimated human trichinellosis cases for pigs from non-controlled housing (Fig. 3). A trend line fitted through the data points characterized the relative risk as *RR* = *69.04* × *%Sensitivity reduction* + *109.8.*

## 4 Discussion

The aim of the present study was to evaluate the infection risk from controlled and non-controlled housed pigs using a modelling approach. The current EU legislation allows exemption from *Trichinella* testing for pigs that are kept under controlled housing conditions, which means that these pigs no longer need to undergo individual carcass control (European-Commission, 2015). We used the previously developed QMRA-T to evaluate the estimated number of human trichinellosis cases under controlled and non-controlled housing condition with- and without *Trichinella* testing at meat inspection. Moreover, we quantified the effect of test sensitivity compared to the gold standard method at meat inspection on resulting human trichinellosis cases per year.

Each year, all EU member states are obliged to report housing type and *Trichinella* test results for all slaughtered pigs to EFSA. Housing types are defined as “controlled housing”, “non-controlled housing” and “not-specified housing”. These data are collated in each member state by the Competent Authorities, but may not be correct in all cases. Obvious errors in the data set were corrected, such as number of pigs slaughtered in a given year, where numbers of tested pools were reported instead of tested pigs as was done for the Netherlands for the year 2015. For this study, data from “not-specified housing” were combined with data from “non-controlled housing”, to not overestimate the number of pigs from controlled housing.

To enable use of the QMRA-T for pigs from controlled housing, we determined a probable number of *Trichinella* larvae in such pigs of at most 80 per 100 g of diaphragm (0.8 LPG). As expected, the sharp increase of modelled *Trichinella* positive pigs around 100 LP100G (on average 1 LPG) could be identified as a sampling effect at this low larval abundance. Indeed, sampling a five times higher weight (5 g instead of 1) increased the probability of finding a positive pig in a pool of animals.

One of the possible risk factors for *Trichinella* transmission to a pig is consumption of a *Trichinella*-infected small mammal breaching bio-security (e.g. a rat entering the pig pens). Depending on the *Trichinella* prevalence in wildlife and the accessibility of *Trichinella* infected wildlife carcasses, small mammals such as rats may be infected at varying levels. The last time a *Trichinella* positive rat was caught in the Netherlands, 0.06 LPG were recorded in one out of 96 brown rats (van Knapen et al., 1993). This would theoretically lead to an infection dose of six larvae if 100 g muscle tissue of this rat were eaten by a pig. Assuming a sex ratio of 70% females and a minimal infectious amount of two *Trichinella* larvae of different sex for rats, and ingestion of 100 g rat muscle tissue by a pig, the majority of such events would not lead to infection (10,000 Monte Carlo simulations) (van der Giessen et al., 2013). Of the pigs that would get infected, around 60% would have a larval abundance up to 10 LPG and some 40% of infected pigs would have 11–110 LPG up to an occasional maximum of 250 LPG in the diaphragm. These levels of infection will not go unnoticed at meat inspection and therefore 0.8 LPG indeed appears a more appropriate hypothetical larval burden for pigs from controlled housing, given the fact that no *Trichinella* positive pigs have been identified from controlled housing where all pigs so far have been tested in the EU. Note however, that the model does not use a given LPG, but larval burden range between 0 and 80 larvae per 100 g.

In our QMRA approach, pigs from controlled housing in the EU represented extremely low risk for humans. With *Trichinella* testing at meat inspection, <0.002 (range 0.000–0.007) residual cases of human trichinellosis per year were estimated, and indeed no human trichinellosis cases in the EU are associated with pork products originating from controlled housing. Without testing, the risk of human trichinellosis was estimated at less than on average 0.010 (range 0.001–0.023) human cases per year, which did not differ significantly. In practice, this means zero human cases per year both with- and without *Trichinella* testing. Thus controlled housing effectively prevents infection risk and *Trichinella* testing for pigs from this housing type does not contribute to food safety. However, not testing for *Trichinella* requires evidence based full compliance with regulations for controlled housing.

In 2014, the FAO/WHO reported residual trichinellosis risk after *Trichinella* testing at meat inspection, expressed as number of infective portions per million servings. In the present study, the residual risk for pigs from controlled housing after *Trichinella* testing ranged 0.00–9.77 × 10^−8^ infective portions per million servings. This is considerably lower than previously estimated (0.017 infective portions per million servings) for 100 million pigs from controlled housing, based on proportional risk estimates (FAO-WHO, 2014b). Moreover, each portion is considered equally infectious to any consumer in the latter study, whereas in the present study, we explicitly determined the variability due to differences in the number of larvae present in infectious portions, as well as difference in consumer susceptibility. To realize this difference in consumer susceptibility, a previously published dose response model for a number of *Trichinella* species (Teunis et al., 2012) had been included in the QMRA-T (Franssen et al., 2017). The output of that dose response model is probability of illness, given exposure to a single portion of undercooked meat and presence of a known number of *Trichinella* muscle larvae in that meat, including the proportion of male and female worms that produce new borne larvae, which cause disease.

Using the QMRA-T, the risk from 80 million pigs that are not kept under controlled housing in the EU was estimated at 832 (95%CI 346–1410) cases per year with testing at meat inspection. This is close to on average 515 annually reported trichinellosis cases, of which 409 have been reported to be confirmed with laboratory tests; non-confirmed cases were based on clinical observations only (EFSA-ECDC, 2009, EFSA-ECDC, 2010a, EFSA-ECDC, 2010b, EFSA-ECDC, 2013, EFSA-ECDC, 2014, EFSA-ECDC, 2015, EFSA-ECDC, 2016). The QMRA-T model results also include cases of mild disease from low numbers of ingested *Trichinella* muscle larvae, whereas reported data from EU member states do not account for mild disease cases. Hence the true number of cases will likely be higher than the epidemiological estimate. On the other hand, the calculated number of portions of pork may have resulted in overestimating the risk, since we modelled all available portions belly, loin and shoulder to be purchased. Probably, part of these portions would also be used for industrialized processing of pork. Without *Trichinella* testing at meat inspection, the number of human trichinellosis cases from pigs from non-controlled housing was 59,443 (95%CI 52,837–66,360). This means that *Trichinella* testing and removal of positive pigs at meat inspection prevents on average 98.6% human trichinellosis cases from non-controlled housing per year for the EU.

The benefits of controlled housing are considerable and quantifiable and not testing pigs from controlled housing seems a logical consequence, which is allowed for the EU internal market under EU Regulation 2015/1375. As a consequence, periodical auditing to demonstrate compliance to regulations regarding controlled housing should be implemented or already be in place, where the choice is made not to test for *Trichinella* (FAO-WHO, 2014a). However, auditing is only cost effective in situations where pigs are produced on a large scale, with relatively low numbers of farms to audit (Alban and Petersen, 2016). Testing 10% of the national pig herd under controlled housing (as included in EU Regulation 2015/1375) seems meaningless, given the minute probability to find a positive pig at an extremely low prevalence (e.g. 1 in 15 million) in such a test sample from controlled housing (Franssen et al., 2017). In low endemic countries like the Netherlands, where also all pigs from non-controlled holding test negative for *Trichinella*, the non-controlled compartment, additional to sows and boars that have to undergo mandatory testing at meat inspection irrespective of housing condition, may be used as sentinel to monitor infection pressure from the environment.

In our view, the QMRA-T model calculated realistic estimates indicating that this model can be of use to evaluate different scenarios. The QMRA-T does not use generally defined test sensitivity, but includes sampling probability from a carcass and the probability of *Trichinella* larva recovery using the artificial digestion test to determine how many *Trichinella* positive pig carcasses will be missed at meat inspection (false negatives). The probability of *Trichinella* larva recovery was based on actual recovery data from proficiency tests including various laboratories and technicians (Franssen et al., 2017). One scenario we investigated is sensitivity of the digestion test at meat inspection relative to the gold standard. We found that a slight decrease in test sensitivity had a significant effect on the number of human trichinellosis cases from pigs kept under non- controlled housing conditions. This may help to identify an acceptable level of performance of a given test in comparison with the gold standard, or to relate consequences for public health to performance of an individual lab using the gold standard.

## 5 Conclusion

We showed that testing 80 million pigs from non-controlled housing for *Trichinella* at meat inspection is preventing on average 98.6% cases of human trichinellosis per year in the EU, compared to not testing. This illustrates the necessity to test pigs from the non-controlled compartment as well as wildlife, to prevent human trichinellosis. We also confirm that for 120 million pigs kept under controlled housing that are slaughtered each year in the EU, *Trichinella* testing does not add health benefits and could be replaced by auditing of farms under controlled management. The QMRA-T can be used to evaluate consequences for public health of different scenarios, such as performance of a given test in comparison with the gold standard.

## Conflict of interest

The authors declare no conflict of interest.
